# A Wireless Brain-Machine Interface for Real-Time Speech Synthesis

**DOI:** 10.1371/journal.pone.0008218

**Published:** 2009-12-09

**Authors:** Frank H. Guenther, Jonathan S. Brumberg, E. Joseph Wright, Alfonso Nieto-Castanon, Jason A. Tourville, Mikhail Panko, Robert Law, Steven A. Siebert, Jess L. Bartels, Dinal S. Andreasen, Princewill Ehirim, Hui Mao, Philip R. Kennedy

**Affiliations:** 1 Department of Cognitive and Neural Systems and Sargent College of Health and Rehabilitation Sciences, Boston University, Boston, Massachusetts, United States of America; 2 Division of Health Sciences and Technology, Harvard University-Massachusetts Institute of Technology, Cambridge, Massachusetts, United States of America; 3 Neural Signals Inc., Duluth, Georgia, United States of America; 4 StatsANC LLC, Buenos Aires, Argentina; 5 Georgia Tech Research Institute, Marietta, Georgia, United States of America; 6 Gwinnett Medical Center, Lawrenceville, Georgia, United States of America; 7 Emory Center for Systems Imaging, Emory University Hospital, Atlanta, Georgia, United States of America; Tel Aviv University, Israel

## Abstract

**Background:**

Brain-machine interfaces (BMIs) involving electrodes implanted into the human cerebral cortex have recently been developed in an attempt to restore function to profoundly paralyzed individuals. Current BMIs for restoring communication can provide important capabilities via a typing process, but unfortunately they are only capable of slow communication rates. In the current study we use a novel approach to speech restoration in which we decode continuous auditory parameters for a real-time speech synthesizer from neuronal activity in motor cortex during attempted speech.

**Methodology/Principal Findings:**

Neural signals recorded by a Neurotrophic Electrode implanted in a speech-related region of the left precentral gyrus of a human volunteer suffering from locked-in syndrome, characterized by near-total paralysis with spared cognition, were transmitted wirelessly across the scalp and used to drive a speech synthesizer. A Kalman filter-based decoder translated the neural signals generated during attempted speech into continuous parameters for controlling a synthesizer that provided immediate (within 50 ms) auditory feedback of the decoded sound. Accuracy of the volunteer's vowel productions with the synthesizer improved quickly with practice, with a 25% improvement in average hit rate (from 45% to 70%) and 46% decrease in average endpoint error from the first to the last block of a three-vowel task.

**Conclusions/Significance:**

Our results support the feasibility of neural prostheses that may have the potential to provide near-conversational synthetic speech output for individuals with severely impaired speech motor control. They also provide an initial glimpse into the functional properties of neurons in speech motor cortical areas.

## Introduction

Perhaps the most debilitating aspect of profound paralysis due to accident, stroke, or disease is loss of the ability to speak. The loss of speech not only makes the communication of needs to caregivers very difficult, but it also leads to profound social isolation of the affected individual. Existing augmentative communication systems can provide the ability to type using residual movement, electromyographic (EMG) signals, or, in cases of extreme paralysis such as that considered here, brain-machine interfaces (BMIs) that utilize electroencephalographic (EEG) signals [1] which can remain intact even in the complete absence of voluntary movement or EMG signals.

A variety of EEG-based BMIs have been used for augmentative and alternative communication (AAC). The main difference between these systems lies in the specific EEG feature used for BMI control. Loosely speaking, the features can be classified as *temporal* (time-series/amplitude based) and *spectral* (frequency based). Common methods based on temporal aspects of the EEG signal include techniques utilizing slow cortical potentials (SCP) [2]–[6] and the P300 visual evoked potential [7]–[12]. Systems utilizing spectral features include techniques involving the sensorimotor rhythm (SMR) [12]–[14] and steady state visual evoked potentials (SSVEP) [15], [16]. Birbaumer and colleagues have developed a communicative BMI called the Thought Translation Device (TTD) [2]–[6] which requires training subjects to first self-regulate the SCP for use in a binary classification paradigm. The resulting control signal is used to actuate binary decision-based augmentative and alternative communication (AAC) devices. Similarly, the Wadsworth Center has developed a spelling program which uses the P300 “oddball” response (visual attention related amplitude increase with 300 ms. latency) to repeatedly select groups of visually presented letters which contain the target letter [[25]–[29,10x]]. The average P300 response and most frequent letter selection are chosen as the desired output. A number of other research groups have begun using the SSVEP as an alternative visual attention-based EEG control feature [15], [16]. The SSVEP is a visual response in which periodically flashing visual stimuli invoke EEG signal frequency components of the same periodicity. SSVEP BMI users can select distinct visually presented targets based on their strobe frequency. Finally, Wolpaw and colleagues have used the SMR signal (μ, β frequency band over sensorimotor electrodes) to control cursor movements on a computer screen in order to select visually displayed letters [12]–[14].

EEG based systems have been used in real-world clinical applications and can provide extremely valuable communication capabilities to profoundly paralyzed users. However they provide only slow verbal or textual output (on the order of one word per minute), making near-normal conversations and social interactions impossible.

In the current study we utilized a novel approach to speech prosthesis that treats the problem as one of speech motor control. We tapped into the speech motor cortical planning circuit by implanting an electrode in speech-related motor cortex (see *Methods* for details) of a 26 year old male volunteer (hereafter the *participant*) who suffers from locked-in syndrome due to a brain stem stroke. Locked-in syndrome is a neurological disorder characterized by intact cognition and awareness but paralysis of nearly all voluntary movement, including speech. The electrode used in the current study was a Neurotrophic Electrode [17], [18] designed for permanent human implantation. Neurites grow into the electrode cone [19], resulting in signaling patterns on the electrode wires within 3–4 months of implantation that are maintained indefinitely (over 4 years in the current participant at date of article submission). Neural signals detected by this electrode were used to drive continuous “movements” of a speech synthesizer that provided audio output to the user in real time. Thus the user received immediate auditory feedback of his ongoing speech that allowed him to improve his utterances with practice.

**Figure 1:**
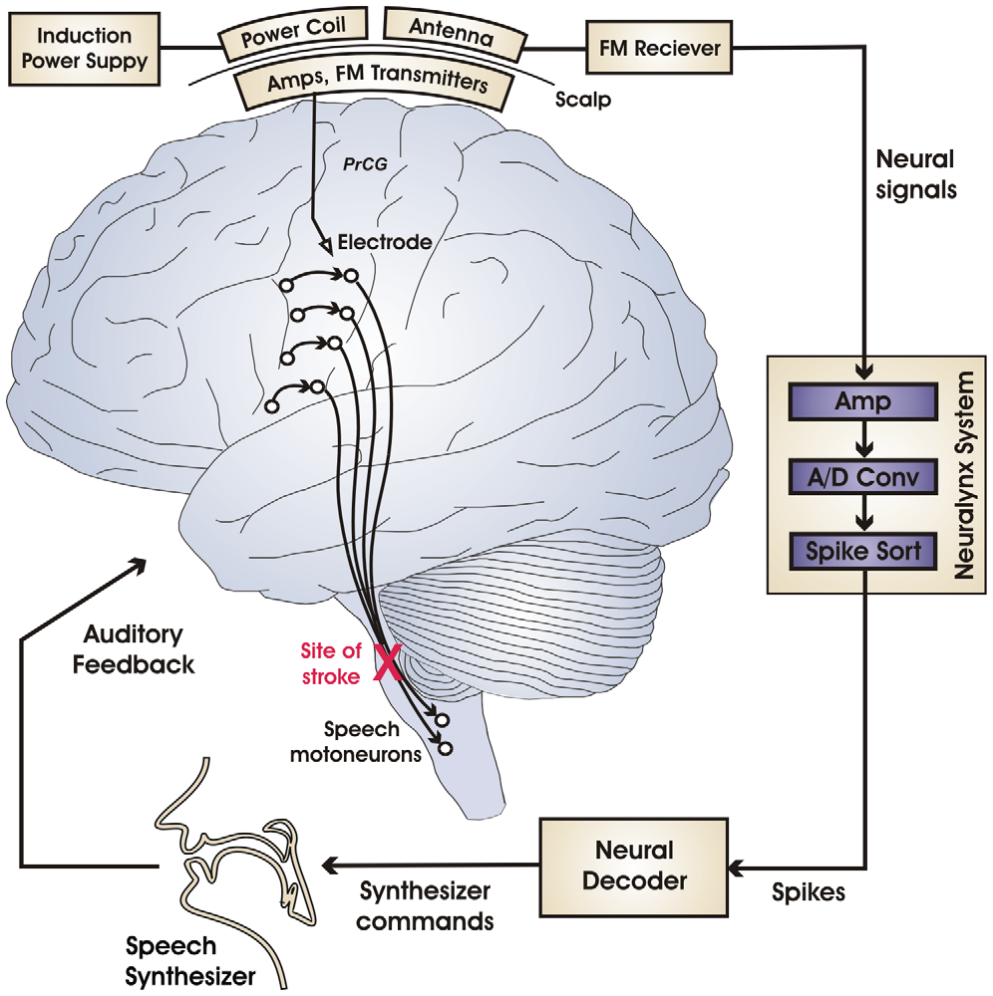
Schematic of the brain-machine interface for real-time synthetic speech production. Black circles and curved arrows represent neurons and axonal projections, respectively, in the neural circuitry for speech motor output. The volunteer's stroke-induced lesion in the efferent motor pathways (red X) disconnects motor plans represented in the cerebral cortex from the speech motoneurons, thus disabling speech output while sparing somatic, auditory, and visual sensation as well as speech motor planning centers in cerebral cortex. Signals collected from an electrode implanted in the subject's speech motor cortex are amplified and sent wirelessly across the scalp as FM radio signals. The signals are then routed to an electrophysiology recording system for further amplification, analog-to-digital conversion, and spike sorting. The sorted spikes are sent to a Neural Decoder which translates them into commands for a Speech Synthesizer. Audio signals from the synthesizer are fed back to the subject in real time. [Abbreviation: PrCG  =  precentral gyrus.]

A schematic of the BMI is provided in Figure 1 (see *Methods* for details). Signals from the two-channel Neurotrophic Electrode are amplified and converted into frequency modulated (FM) radio signals for wireless transmission across the scalp. This telemetric system eliminates the need for wires or connectors passing through the skin, allowing permanent implantation without risk of infection. During data collection, the subcutaneous electronics are powered by an induction power supply via a power coil temporarily attached to the subject's head using a water-soluble paste. Two additional coils act as receiving antennae for the FM signals. The signals are then routed to a Neuralynx Cheetah electrophysiological recording system that digitizes them and performs spike sorting. The sorted spikes are then sent to a Neural Decoder whose output constitutes the input to a speech synthesizer that provides the subject with audio feedback. The neural decoder and speech synthesizer are implemented on a desktop computer running Windows XP. The delay from neural firing to corresponding audio output is 30–70 ms with an estimated average delay of 50 ms, approximating the delay from motor cortical activity to corresponding speech sound output in the neurologically intact human.

**Figure 2:**
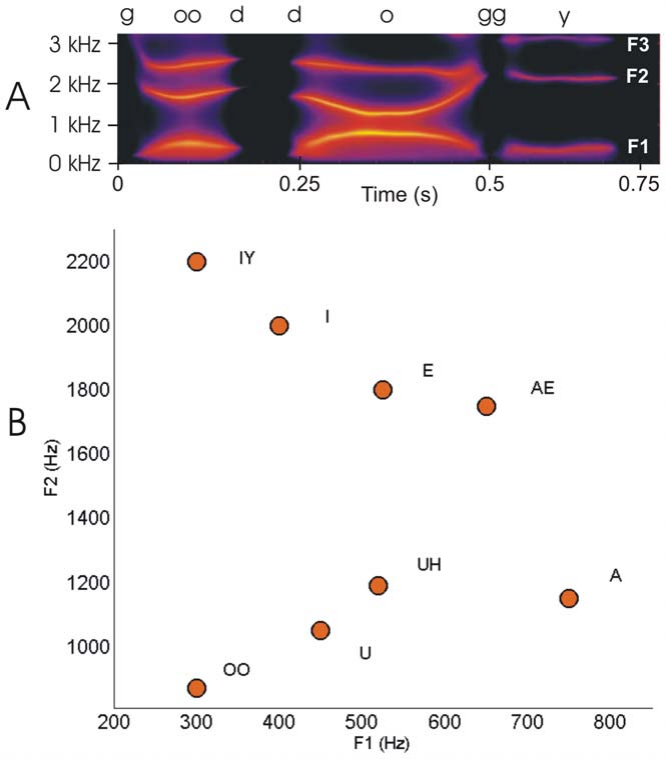
Formant frequencies in speech. (A) Spectrogram of the utterance “good doggy” with the trajectories of the first three formant frequencies (F1, F2, F3) clearly visible as bright bands of high energy. (B) Approximate locations of the monophthongal vowels of English plotted on the plane formed by the first two formant frequencies.

According to an established neurocomputational model of speech motor control [20], [21], neurons in the implanted region of left ventral premotor cortex represent intended speech sounds in terms of *formant frequency trajectories*, and projections from these neurons to primary motor cortex transform the intended formant trajectories into motor commands to the speech articulators. Formant frequencies take on continuous values corresponding to the locations of peaks in the spectral envelope of the acoustic signal (see Figure 2A), which in turn are related to the spatial positions of the speech articulators [22], much like the spatial position of a fingertip relative to the body is determined by joint angles of the arm, wrist, and hand. Carrying this analogy further, speech can be characterized as a “movement trajectory” in formant frequency space (in the current case, a 2-dimensional space characterized by F1 and F2) much like a reach can be described as a 3-dimensional movement trajectory of the fingertip in Cartesian space. The sounds of speech can be identified from the trajectories of the formant frequencies across time, even in the absence of other speech cues [23]. Formants thus provide a meaningful, low dimensional representation of both speech acoustics and the vocal tract configuration. Formant frequencies are also a convenient choice for real-time speech synthesis since formant-based synthesis has been studied for decades [24]–[27] and synthesizer software that requires very little computational time is available.

Here we focus on vowels, which can be specified by target values of the first two formant frequencies. Figure 2B shows the locations of the monophthongal vowels of English in the *formant plane* defined by the first formant frequency F1 (represented on the x axis) and the second formant frequency F2 (represented on the y axis). A vowel-to-vowel speech movement such as UH-IY can be represented by a line from one vowel location (corresponding to UH) to another (corresponding to IY) in this plane; this line indicates the *formant trajectory* for producing UH-IY in the F1/F2 plane. The current system utilizes a Klatt-based formant synthesizer [28] with all parameters set to fixed values except F1 and F2, which were determined by the output of the Neural Decoder.

## Results

### Formant Frequency Analysis

To determine whether the recorded signals contained information regarding intended formant frequencies during speech (or in the current case, attempted speech), statistical analyses were performed on normalized spike data collected during eight 63.4 s long attempted speech sessions recorded on separate days. During each session, the participant was asked to attempt to speak in synchrony with a vowel sequence stimulus that was presented auditorily. The sequence consisted of 1-second long steady state vowels (UH, IY, A, or OO) with 300 ms linear formant transitions between vowels, and the vowel sequence was highly predictable to facilitate near-synchronous imitation. No audio or visual feedback of his performance was provided. Neural data collected during the production attempt were time-aligned with the formant frequency trajectories of the stimulus being mimicked, and ridge regression analyses were used to determine optimal linear fits of the intended formant frequencies from the firing rates of 56 spike clusters (see Methods for spike sorting details). We will use the term “units” to describe these clusters with the understanding that they represent a mix of single- and multi-unit clusters.

We first tested the hypothesis that the firing rates collectively encode information regarding the first and second formant frequencies of the intended speech utterance. Specifically, the correlation coefficient between each formant frequency (F1 and F2) and the optimal linear combination of all firing rates was estimated using two-fold cross-validation within each session, and the resulting coefficients were then averaged across sessions. Significant correlations were found for both F1 (r = 0.49, p<0.001) and F2 (r = 0.57, p<0.001), thus confirming the hypothesis.

**Figure 3:**
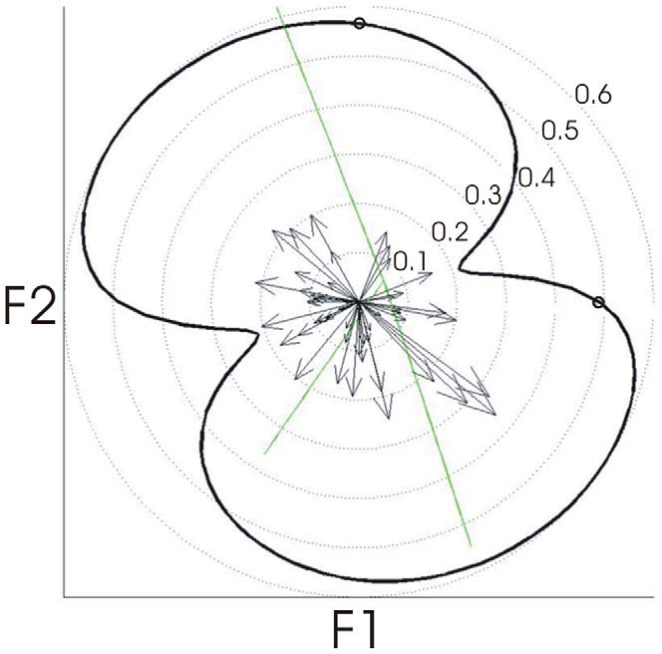
Formant tuning of individual units and of the neural ensemble. **Individual units**: Black arrows represent the formant tuning of individual units in polar coordinates, with angle representing the preferred direction of movement and arrow length representing the tuning strength (see Methods for details). The strength of tuning to each possible direction of movement in formant space is computed as the average correlation (across sessions) between each unit's firing rate and the target formant position along this direction. The preferred direction of movement is then computed as the direction with maximal tuning strength among all possible directions. **Neural ensemble**: The black curve represents the formant tuning of the neural ensemble in polar coordinates, with angles representing each possible direction of movement and distance from origin representing the tuning strength. Green lines represent the directions of movement in formant space of the three target sounds used for training, and the two small circles along the neural ensemble tuning curve represent the average strength of the correlations between firing rates and F1 (r = 0.49, p<.001), and F2 (r = .57, p<.001), respectively.

**Figure 4:**
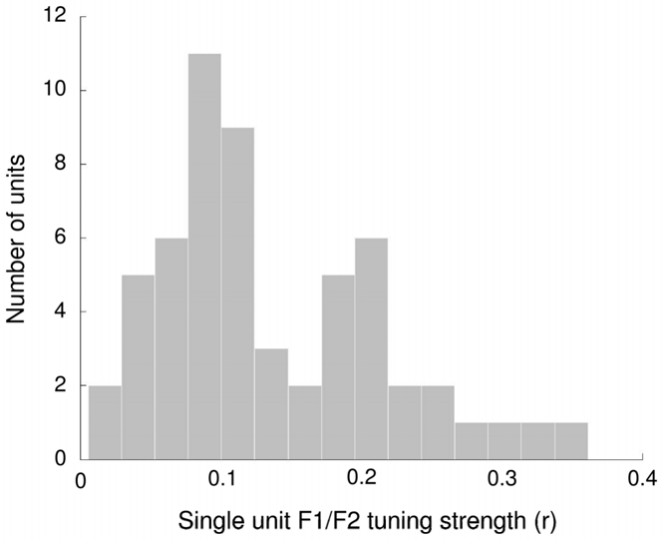
Distribution of unit correlation coefficients (indicative of formant tuning strengths in the unit's preferred direction) averaged across sessions. The first peak is representative of units with “poor” tuning, the second peak represents units with “average” tuning, and the right distribution tail represents units with “good” tuning.

**Figure 5:**
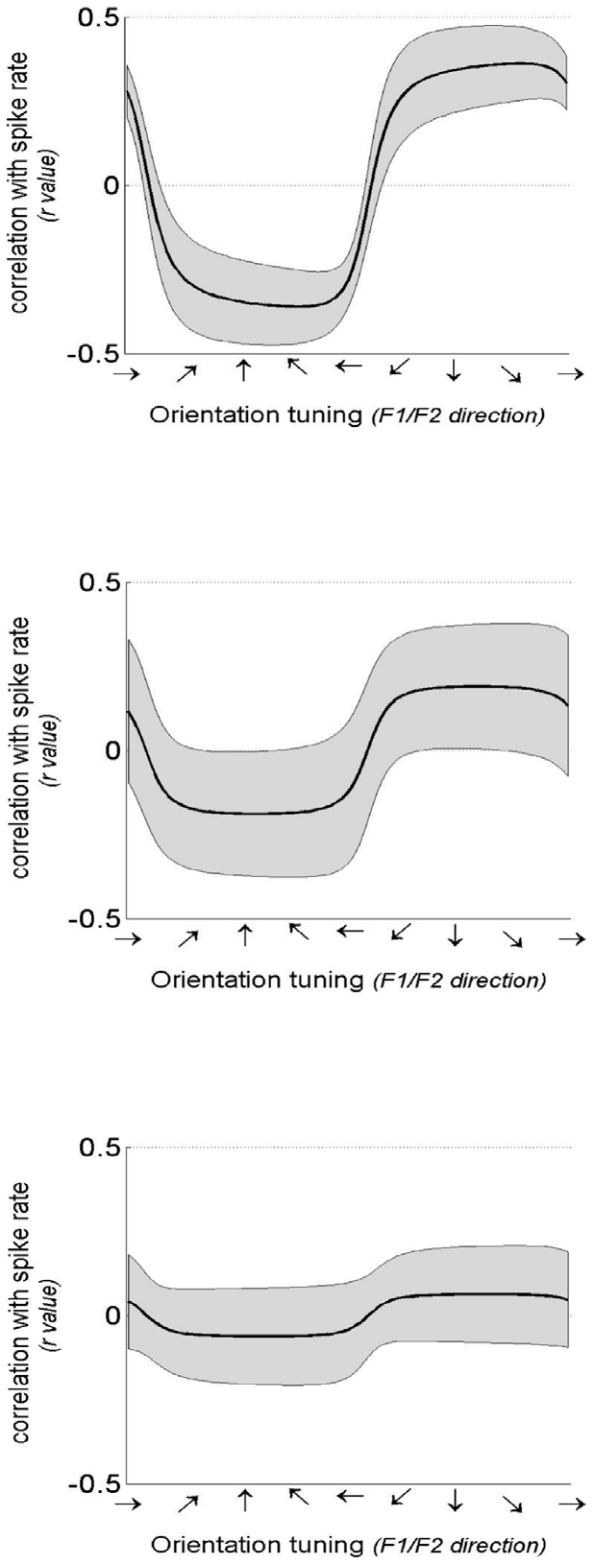
Sample tuning curves for representative good (top), average (middle), and poor (bottom) tuned units. Tuning curves (black) and 95% confidence intervals (gray) are computed as described in *Methods* *: Formant tuning analyses*. Tuning strength is indicated by the correlation between unit firing rates and formant frequency. The three units shown are primarily tuned to changes in F2, illustrated by relatively low values at each horizontal direction.

This analysis was then generalized to test for tuning preferences to all movement directions in formant space (rather than just along the F1 and F2 axes). Results of formant tuning analyses of individual units are presented in Figure 3. Of the 56 units identified, 24 (43%) showed statistically significant tuning strengths across sessions (i.e., average correlation greater than zero; p<.05) and relatively stable preferred directions (95% confidence intervals within the same half-plane). Tuning strengths among these units ranged between r = .10 and r = .36. The overall distribution of correlation coefficients for individual units is shown as a histogram in Figure 4. The main modes of the distribution represent the “poor” and “average” tuned units while the upper tail represents units with “good” tuning. Example tuning curves for representative units with good, average, and poor tuning are shown in Figure 5. The average correlation coefficient is shown on the y-axis and the preferred direction on the x-axis. The 95% confidence intervals shown were estimated via random sampling with replacement across sessions.

Analyses of the neural ensemble also showed significant tuning strengths among all the directions of movement in the F1–F2 plane (p<.001; with tuning strengths ranging between r = .21 and r = .60). The neural ensemble as a whole showed stronger tuning (black curve in Figure 3) for directions aligned approximately along the long axis of the experimental formant target distribution (green lines in Figure 3; maximum distance for any individual session 58°, p = .005). Furthermore, the ensemble preferred direction was found to be stable across sessions (s.d. of the preferred direction axis was 26°).

**Figure 6:**
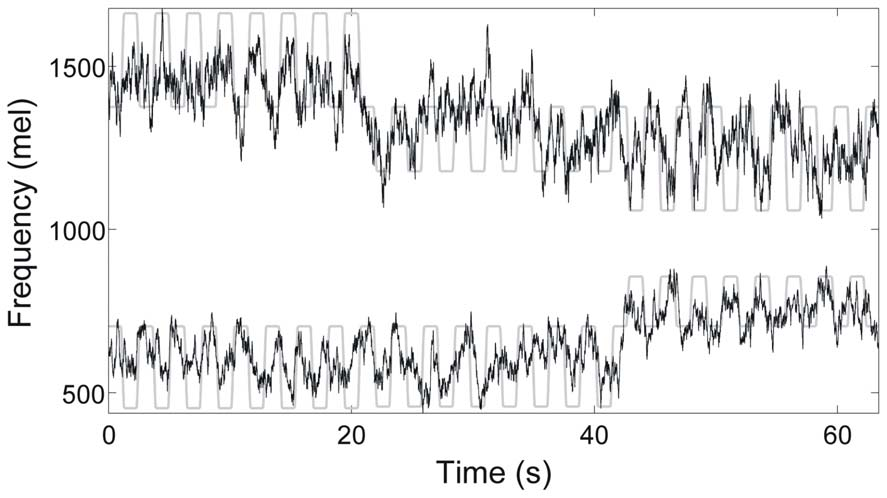
Results of offline ridge regression reconstruction of intended formant frequencies while the participant attempted to speak in synchrony with a speech stimulus. Reconstructed values of the first (bottom) and second (middle) formant frequencies (in Mel units) are shown (black lines) along with the formant frequencies present in the stimulus being mimicked (gray lines).

Figure 6 shows the reconstructions of intended formant frequencies for one recording from an offline ridge regression fit (ridge parameter set to .01; whole dataset used for illustration purposes). Gray lines represent the F1 and F2 trajectories (measured in Mel units) of the target stimulus. Black lines represent the ridge regression fit (r = .69 for F1; r = .68 for F2).

In sum, the results from the offline formant frequency analyses support the DIVA model's prediction that neurons in the implanted area carry information regarding the intended formant frequency trajectory during speech production (attempted speech, in this case).

### Real-Time Feedback Sessions

To investigate whether the participant could learn to use the real-time BMI to improve his production of speech sounds, 25 real-time feedback sessions were carried out over a 5-month period. Prior to the start of each real-time feedback session, a Kalman filter was trained to predict intended formant frequencies from unit spiking rates collected in the 63.4-second vowel sequence imitation protocol described above. This process is largely analogous to that used in prior Kalman filter-based cursor control BMI studies [29], [30]. The resulting Kalman filter was implemented in the Neural Decoder of Figure 1. Each real-time session involved 5–34 attempted productions of vowels using the BMI, broken into 1–4 blocks interspersed with short rest periods. Each production trial started with the formant synthesizer in a central vowel location (corresponding to the vowel UH, as in “hut”) and required the participant to change the sound (by changing the neural signals that drive the BMI) to a peripheral vowel location within six seconds. The target vowel was IY (as in “heat”), OO (as in “hoot”), or A (as in “hot”) depending on the trial. This task can be viewed as a “center out task” carried out in the two-dimensional formant frequency space defined by F1 and F2.

**Figure 7:**
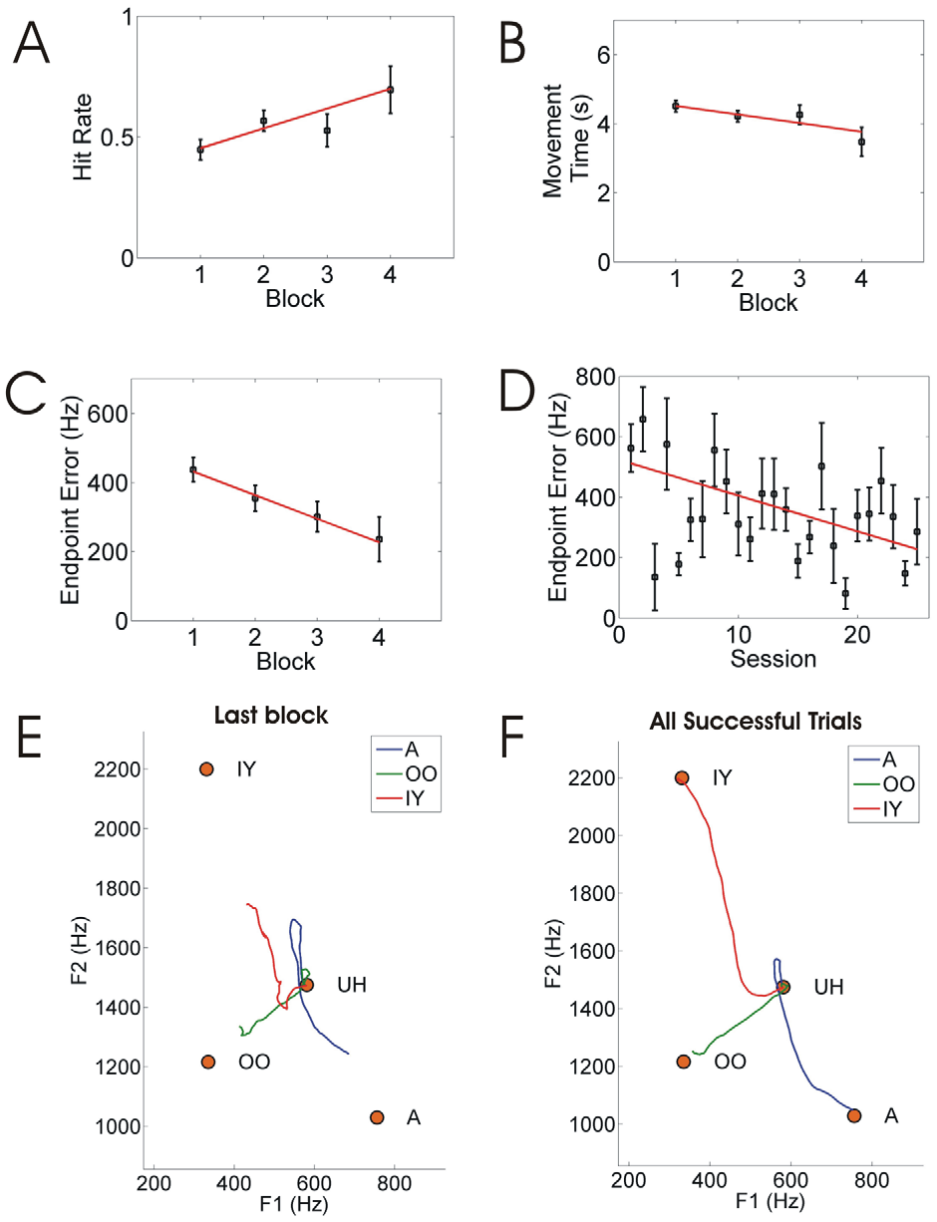
Results for vowel production task with real-time synthesizer. (A–C) Performance measures as a function of block number within a session, averaged across all sessions: (A) hit rate, (B) movement time, and (C) endpoint error. (D) Average endpoint error as a function of session number. (E–F) Average formant trajectories for utterances in (E) the last block of all sessions, and (F) successful trials in all blocks and sessions.

Results of the 25 real-time feedback sessions are provided in Figure 7. Panels A–C indicate hit rate, mean movement time, and mean endpoint error (measured as the difference between the formant space position at the end of the trial and the target formant position for that trial) as a function of block number, averaged across all 25 sessions. All three performance measures improved significantly (p<0.05; t-test with null hypothesis of zero slope as a function of block number). Figure 7D shows average endpoint error as a function of session number. Average endpoint error decreased significantly over the 25 sessions, indicating that the participant improved his performance across days as well as within a session.

Panels E-F in Figure 7 show average formant frequency trajectories produced by the subject for each target vowel. Because the duration of experimental trials varied depending on time of successful target acquisition, the individual trial formant frequency trajectories were time normalized using dynamic time warping [31] and averaged to produce the trajectories indicated in the figure. Panel E shows average trajectories for IY, A, and OO during the last block of each session, averaged across sessions. Panel F shows the average trajectories on the correct trials across all blocks and sessions. Supplementary Video S1 illustrates the participant's performance on five consecutive vowel production trials.

## Discussion

Our results demonstrate the ability of a profoundly paralyzed individual to relatively quickly (over the course of a 1.5 hour session involving 34 or fewer vowel production attempts) improve his performance on a vowel production task using a BMI based on formant frequency estimates decoded in real-time from the activity of neurons in his speech motor cortex. The participant's average hit rate rose from 45% on the first block to 70% on the last block across sessions (reaching a high of 89% in the last block of the last session) and average endpoint error decreased from 435 Hz to 233 Hz from the first to the last block across sessions. These results generalize previous findings of successful motor learning for control of a computer cursor by paralyzed humans with implants in the hand/arm area of motor cortex [29], [30], [32]–[34], an ability previously demonstrated in monkey BMI studies [35]–[37], to the domain of speech production. They also support the feasibility of neural prostheses that could decode and synthesize intended words in real-time, i.e. as the (mute) speaker attempts to speak them.

Though the abilities of the current participant are limited to producing a small set of vowels, it is noteworthy that this performance was possible using only a single 3-wire electrode. Findings from monkey BMI studies indicate that large performance improvements can be achieved with more recording sites and decoding techniques that co-adapt with the user to provide faster learning [38]. In the long term, we believe that implementation of such improvements to BMI systems like the current one should allow rapid, accurate control of a synthesizer that can produce consonants as well as vowels [20], [39] with high accuracy. Current augmentative communication devices for profoundly paralyzed patients, including non-invasive BMIs based on detection of event related potentials (ERP) using electroencephalography [2]–[5], rely on a relatively slow typing process (approximately 1–6 characters per minute at a minimum 70% accuracy level for production of intended words in amyotrophic lateral sclerosis [5] and paraplegic [6] patients). In contrast, real-time decoding of intended auditory or articulatory parameters for control of a speech synthesizer holds the promise of allowing the production of words at near-normal conversational rates.

The current work also constitutes, to our knowledge, the first successful use of a permanently installed, telemetric implant (requiring no wires or connectors passing through the skin, thereby greatly reducing the risk of infection) for real-time control of an external device. Such wireless systems, combined with permanently installed electrodes such as the Neurotrophic Electrode, constitute an important milestone in the development of safe, permanent neural prostheses for profoundly paralyzed individuals that require no major external hardware beyond a wireless receiver and laptop computer.

Finally, our results provide a rare glimpse at how neural ensembles in the brain encode intended speech utterances. Unlike motor tasks such as reaching and drawing, which have been studied extensively in electrophysiological investigations in primates [40]–[42], speaking is a uniquely human attribute that has to date eluded investigation at the neuronal level. In the intact brain, movements of the speech articulators are generated by activity in motor cortex via motoneurons in the brain stem [43]. Motor cortical neurons are themselves activated by neurons in adjoining premotor cortex that are responsible for planning the speech movements needed to produce the currently intended speech utterance. The electrode in the current study was located near the boundary between premotor and primary motor cortex for the speech articulators. In the domain of arm/hand movements, many motor and premotor cortical neurons have been shown to code movement direction of the hand; that is, neural firing rates correlate strongly with movement direction in Cartesian space in both monkeys [40], [41] and humans controlling a computer cursor [44]. To our knowledge, there have been no prior electrophysiological reports of neuronal firing rates in speech motor areas. This situation forced us to rely on a neurocomputational model of speech motor control for insight into neural coding properties in these areas. The DIVA model of speech production [20] posits that neurons in left ventral premotor cortex encode desired formant frequency trajectories, which are then mapped into articulator movements in the primary motor cortex. Cross-validated ridge regression analyses indicated significant correlations between unit spiking rates and intended formant frequencies of speech utterances, supporting the DIVA model prediction.

Since formant frequencies are closely related to positions of the speech articulators, this finding suggests that it should be possible to decode intended positions of the speech articulators from these same signals. This is analogous to electrophysiological findings indicating the encoding of cursor movement as well as hand/arm movement in motor cortical neurons of monkeys trained to control movements of a computer cursor [45]. Uncertainties regarding intended positions of the speech articulators in the current study (due to complete lack of movement capabilities in the subject) prevent a thorough assessment of this possibility here. A potential advantage of decoding articulator positions, rather than formant frequency values, is that consonant production should be easier using a low degree-of-freedom articulatory synthesizer, such as a modified version of the 7-dimensional Maeda articulatory synthesizer [39], than with a formant synthesizer. This is because realistic-sounding stop consonants can be produced with relatively slowly varying control signals that move an articulator toward a closure of the vocal tract in an articulatory synthesizer, whereas in a formant synthesizer rapid control of several parameters is required to achieve realistic-sounding consonant closures. In ongoing work we are developing a low-dimensional articulatory synthesizer for use in the BMI.

## Methods

### Ethics Statement

The implantation procedure was approved by the Food and Drug Administration (IDE G960032), Neural Signals, Inc. Institutional Review Board, and Gwinnett Medical Center Institutional Review Board, and all experimental procedures were approved by the Neural Signals, Inc. Institutional Review Board. Informed consent was obtained from the participant (via eye-blink signaling, the only form of communication available due to near-complete paralysis) and from his legal guardian in writing for all procedures prior to the study.

### Participant

The 26 year old male participant in the current study suffers from locked-in syndrome due to a brain stem stroke incurred at age 16, leaving the brain areas responsible for consciousness, cognition, and higher-level aspects of movement control intact while eliminating nearly all voluntary movement. The participant's voluntary motor output was limited to slow vertical movements of the eyes, allowing him to answer yes/no questions. Audition and somatic sensation were not noticeably impaired, and visual perception was largely intact except for an inability to control the eyes in a conjugate fashion, resulting in an inability to foveate and/or track objects or locations of interest. Implantation occurred at age 21, approximately 5 years after becoming locked-in due to brain stem stroke and 38 months prior to the start of the current study.

### Implant Localization

**Figure 8:**
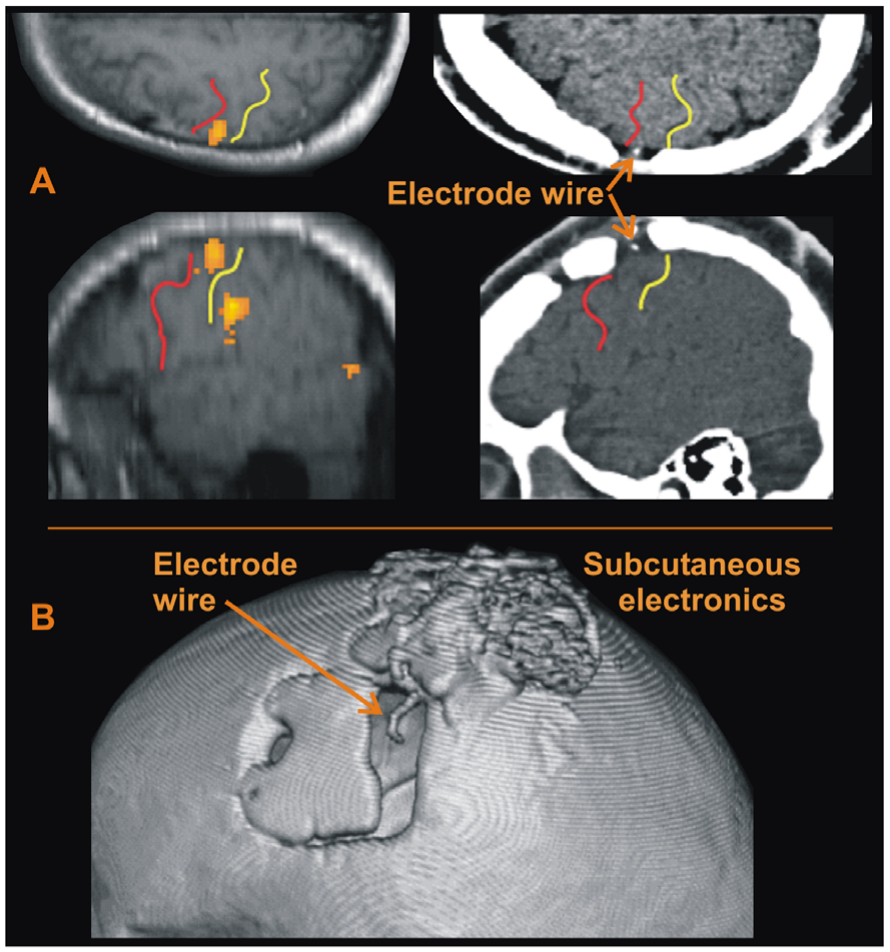
Electrode location in the participant's cerebral cortex. (A) Left panels: Axial (top) and sagittal (bottom) slices showing brain activity along the precentral gyrus during a word generation fMRI task prior to implantation. Red lines denote pre-central sulcus; yellow lines denote central sulcus. Right panels: Corresponding images from a post-implant CT scan showing location of electrode. (B) 3D CT image showing electrode wire entering dura mater. Subcutaneous electronics are visible above the electrode wire, on top of the skull.

The implant location was chosen based on the results of a pre-surgery functional magnetic resonance imaging (fMRI) scan. Images were collected with a 1.5 Tesla scanner while the subject attempted to perform a picture naming task. Activity during picture naming was contrasted with a baseline condition of quiet rest, and the implant location was chosen as a region of peak activity on the precentral gyrus (see Figure 8). The electrode was implanted at the identified location using MRI-guided stereotactic surgery.

### Signal Acquisition

Extracellular voltage potentials recorded by the Neurotrophic Electrode are on the order of 10–50 µV and are 100x gain amplified by subcutaneous hardware before being transcutaneously transmitted by wireless FM radio. Once received, the potentials are again 100x gain amplified. The potentials on each channel are then sampled by a Neuralynx, Inc. Cheetah data acquisition system at 30303 Hz where the signal is split into two processing streams, with one set of signals lowpass filtered at 9000 Hz and the other bandpass filtered between 300 Hz and 6000 Hz. Both data sets are recorded onto digital tape and sent to the Cheetah acquisition software where they are committed to file. In addition to storing the signals to disk, the Cheetah software performs real-time spike sorting on the bandpass-filtered data stream (described further below); the output of these procedures is stored to disk and, during real-time feedback trials, sent to the computer which performs neural decoding and speech synthesis.

### Spike Sorting

A positive and negative voltage threshold detector was applied to the bandpass filtered signals to determine the arrival time of putative action potentials and a 32-sample (1.05 ms) spike waveform was saved for further analysis. The detection threshold was set to ±10 µV (the non-neurophysiological noise level of the wireless electronics system) and all spikes were aligned to the 8^th^ sample (∼0.25 ms). Spike clusters were defined by visual inspection using a convex-hull method (SpikeSort3D Neuralynx Inc., Bozeman, MT) along a five-dimensional feature vector including: 1) peak and 2) valley amplitude, 3) spike height, 4) spike waveform energy and 5) the 8th sample amplitude. The primary decision feature was spike peak and valley amplitude (depending on the initial spike deflection based upon the 8th sample amplitude). Inter-spike interval histograms and auto-correlograms were used to describe the firing characteristics of each spike cluster, and cross-correlograms indicated interactions between clusters. The spike clusters were refined by splitting cluster regions along the specified feature dimensions until the split clusters showed increased cross-correlation. Increases in cross-correlation were taken as an indication that the split clusters belonged to the same parent cluster. The final cluster boundaries were set as the last convex-hull definition prior to any increase in cross-correlation. According to this method a total of 29 clusters were defined from the multi-unit signal on the first recording wire and 27 clusters on the second for a grand total of 56 distinct clusters. These numbers are overestimates of the number of single unit clusters (i.e., clusters whose spikes come from a single neuron). In particular, many of these clusters represent the combined signals of multiple neurons (i.e., multi-unit activity). We chose to err on the side of overestimating the number of clusters in our BMI since our Kalman filter decoding technique is somewhat robust to noisy inputs, whereas a stricter criterion for cluster definition might leave out information-carrying spike clusters.

### Global Normalization and Smoothing

The firing patterns for each cluster as determined above were next processed by a global normalization and smoothing procedure designed to factor out slow fluctuations in the total neural activity (across all clusters) that could occur over the course of an experimental session or across sessions. More generally, this normalization procedure, detailed in the following paragraphs, was designed to yield firing rate estimates that were robust over wide ranges of spiking activity.

The normalized firing rate for each unit was determined by convolution of an adaptive smoothing filter with the spike train modeled as a series of delta functions. The smoothing filter was defined as causal (thereby allowing use in a real-time system), compact and smooth. A suitable filter is the exponential function given in general form as:
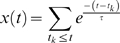
where 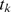 denotes spike arrival time, k indexes sequentially the detected spikes, and 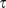 is an exponential decay parameter. The exponential probability function is a theoretically suitable model for the waiting time distribution of a Poisson process [46], which is commonly taken as a first order model of neural spike trains. Here we define an adaptive exponential filter optimized for the current application.

Two steps were utilized to achieve firing rate normalization. The first was to allow the exponential decay parameter to vary slowly over time according to:
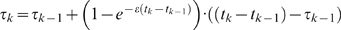
where 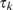 is the adaptive decay rate and 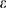 controls the rate of adaptation. The effect of this equation on the exponential filter in the first equation is to increase the filter size (in time) when the baseline spiking activity is low and to decrease it under high activity scenarios.

The second step was to use a log transform in order to transform exponential data into Gaussian distributed data. The final log-transformed adaptive exponential filter is given by:
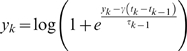


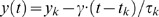
where 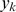
is the estimated firing rate at the time of the k^th^ spike arrival, and *y(t)* is the estimated firing rate at time *t* (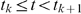). A final parameter 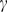 is a scaling factor for the filter. The two parameters effectively perform a band-pass filtering of the estimated firing rate with 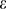 as the high-pass and 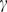 as the low-pass. The values used for these parameters were determined by an initial optimization on the regression between the normalized firing rates and the target formant trajectories.

### Formant Tuning Analyses

In order to analyze the association between firing rates and formant frequencies, the target formant trajectories were projected along one hundred 1-dimensional axes with angles equally spaced between 0 and 2π in the F1–F2 plane. These projected trajectories were used to estimate the preferred direction and tuning strength of each unit's response in isolation as well as of the neural ensemble. For individual units, the correlation coefficient between each of these projected trajectories and each identified unit's firing rate was computed within each session and then averaged across sessions. The maximum average correlation across the 100 formant directions was used to define the neuron's *preferred direction* (direction of movement maximizing the average correlation) and *tuning strength* (average correlation coefficient at the preferred direction). Random sampling with replacement across the eight experimental sessions was used to estimate 95% confidence intervals and statistical significance of each parameter estimate.

Typically, choosing the maximum average correlation across sessions requires a multiple comparison correction (e.g., Bonferroni). However, the 100 measures computed in the individual unit tuning analysis show high degrees of *dependence* between one another relative to the *independence* assumed in a Bonferroni correction. In fact, the maximum average correlation coefficient empirically estimates the direction obtained via multivariate regression between the firing rates and the two-dimensional formant frequency trajectory. As such, we estimate that the effective Bonferroni correction is approximately 2 rather than 100 (which would be the case assuming that all 100 measures were completely independent). We confirmed this by Monte Carlo simulation using random firing rates and random F1–F2 trajectories conforming otherwise exactly to the methods followed in the manuscript. The observed false-positive rate under the null hypothesis was 9.5% (c.f. an expected 5%) after 1000 simulations when using a p<0.05 threshold. This result confirms the effective dimensionality of the multiple comparisons as approximately 2. From this perspective the selection criteria is conceptually similar to selecting those units that are significantly associated with either F1 or F2 trajectories. We opted to report results utilizing the uncorrected p<.05 false-positive rate (corrected p<.095) as an appropriate compromise between sensitivity and specificity when exploring the contribution of individual units to the neural ensemble.

To analyze the entire neural ensemble, the correlation coefficient between each of these projected trajectories and the optimal linear combination of all neuron's firing rates was estimated using two-fold cross-validation within each session, and the resulting coefficients were then averaged across sessions. Within each session, ridge regression was used on half of the data in order to estimate the linear combination of the neurons' firing rates best fitting each projected formant trajectory. The correlation coefficient between the obtained linear combination of firing rates and projected formant trajectories was then computed on the second half of the data. This procedure was repeated for each formant projection, and the resulting cross-validated correlation coefficients were averaged across sessions, defining the ensemble's formant tuning function. Random sampling with replacement across sessions was used to estimate confidence intervals and statistical significance of each parameter estimate.

To analyze the ensemble's tuning direction, the direction at which the ensemble's formant tuning function achieved its maximum was defined as the ensemble's preferred direction. Monte Carlo simulations were used to investigate the possible association between the ensemble's preferred direction and the principal axis (first principal component) of the distribution of formant targets used in these experiments. One thousand artificial session data were created using simulated firing rates drawn from a white noise distribution. For each simulated dataset the same formant target trajectories were used as in the real experiments, in order to accommodate any possible bias that this distribution could impose on the resulting formant tuning curves unrelated to the neuron's firing rates. For each simulation the angular distance between the ensemble's preferred direction and the principal axis of the distribution of formant targets was computed. The mean and maximum angular distances across random sets of eight sessions were computed and their distributions estimated and compared to the equivalent measures obtained from the eight real experimental sessions.

### Kalman Filter for Real-Time BMI

A static Kalman filter [29], [47] was used for decoding the normalized neural signals in the real-time BMI. Specifically, the Kalman filter was used to predict the intended positions and velocities of the first two formant frequencies based on the current values of the normalized neural firing rates. A Kalman filter is a linear Gaussian model (LGM) in which both external states and likelihoods (i.e. relationship between formant frequencies and neural activity) are explicitly modeled. These two components are given by:
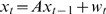


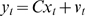
where 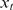 is the vector of formant frequency positions and velocities and 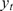 is the vector of neural activity at time 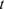. The matrix 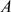 describes the relationship between past and future formant frequencies while 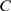 describes the expectation of neural activity given a set of formant frequencies. Both are determined by least-squares linear regression. The error terms 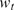 and 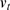, are Gaussian random variables 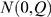 and 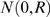 respectively with 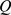 the residual covariance of the state linear dynamical system and 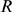 the residual covariance of the likelihood function.

Values for the Kalman filter parameters were obtained at the beginning of each recording session using training data collected during the one-minute vowel sequence mimicry protocol described above. Only units with significant functional relationship (multiple linear regression F-test, p<0.05) to the intended speech sequence were used during any particular session. The Kalman filter decoder was written as a C++ dynamic library utilizing static state transition and generative function parameters. The computational overhead of the static Kalman filter was well within tolerances for a real-time system; a single update step of the Kalman filter library required less than 1 ms of computational time.

### Speech Synthesizer

Real-time audio feedback was achieved by instantaneous computer speech synthesis of predicted formant frequencies from the Kalman filter. Specifically, a C-language implementation of the Klatt [28] formant-based speech synthesizer was used for speech synthesis. The formant synthesizer utilizes a total of 63 user-specified parameters, of which only two, corresponding to the first and second formant frequencies (F1 and F2), were actively controlled by the participant. The remaining parameters, whose values affect sound quality and naturalness [48], were fixed to typical values. The computational overhead of the speech synthesizer was very low, requiring less than 1 ms of computational time to synthesize a 10 ms sound waveform. Synthesized waveforms were directly buffered onto the onboard soundcard via the DirectSound interface and played on speakers positioned in front of the participant.

### Real-Time Vowel Production Task

The real-time experimental task was based on the “center-out” design used in many motor control studies. Each trial of a center-out task involves movement from a central location to a target location randomly chosen from a set of peripheral target locations. For example, a computer cursor may be moved by a mouse from a central location on a screen to one of eight peripheral targets [29]. In the current case, the center-out task was carried out in an auditory space defined by the formant frequency plane, involving movement from a central vowel location (UH in Figure 2B) to one of three peripheral vowel locations (IY, A, OO in Figure 2B). The three target vowels are located at three extreme corners of the F1/F2 space for English vowels. The target stimulus was randomly chosen on each trial and was presented acoustically to the subject prior to the start of the production attempt. After target stimulus presentation, the subject was given an instruction to “speak” the recently heard stimulus. After the speak instruction, the BMI began synthesizing formant frequencies predicted from the current neural activity. The trial was ended after a maximum duration of 6 seconds or when the decoded formant frequencies entered and remained inside a target region around the endpoint vowel for 500 ms. These circular target regions spanned approximately 150 Hz in F1 and 300 Hz in F2 and contained a small attractor force to help keep the participant's production within the target region once it was entered. This attractor force did not affect the formant trajectory outside the target regions. For the first 10 sessions, no visual feedback was provided to the subject. In the last 15 sessions the subject could view a cursor position in the formant plane corresponding to the ongoing sound output. No difference in performance was noted between sessions with visual feedback and sessions without visual feedback.

## Supporting Information

Video S1 Supplementary Video S1 illustrates the participant's performance with the BCI during consecutive five trials in a real-time feedback session. Each trial starts with the computer playing the word “listen” followed by the utterance to be produced (a vowel-to-vowel utterance starting at the vowel UH and moving to the target vowel for that trial). The computer then plays the word “speak”, which is followed by the subject's attempt to produce the utterance with the BCI, including the synthesizer sound output and corresponding formant plane representation. A successful attempt occurs if the cursor reaches the target vowel location (indicated in green) within six seconds.
(8.85 MB MOV)
